# Metabolic Compensation of Fitness Costs Is a General Outcome for Antibiotic-Resistant *Pseudomonas aeruginosa* Mutants Overexpressing Efflux Pumps

**DOI:** 10.1128/mBio.00500-17

**Published:** 2017-07-25

**Authors:** Jorge Olivares Pacheco, Carolina Alvarez-Ortega, Manuel Alcalde Rico, José Luis Martínez

**Affiliations:** Centro Nacional de Biotecnología, CSIC, Madrid, Spain; University of British Columbia

**Keywords:** *Pseudomonas aeruginosa*, antibiotic resistance, efflux pumps, fitness costs

## Abstract

It is generally assumed that the acquisition of antibiotic resistance is associated with a fitness cost. We have shown that overexpression of the MexEF-OprN efflux pump does not decrease the fitness of a resistant *Pseudomonas aeruginosa* strain compared to its wild-type counterpart. This lack of fitness cost was associated with a metabolic rewiring that includes increased expression of the anaerobic nitrate respiratory chain when cells are growing under fully aerobic conditions. It was not clear whether this metabolic compensation was exclusive to strains overexpressing MexEF-OprN or if it extended to other resistant strains that overexpress similar systems. To answer this question, we studied a set of *P. aeruginosa* mutants that independently overexpress the MexAB-OprM, MexCD-OprJ, or MexXY efflux pumps. We observed increased expression of the anaerobic nitrate respiratory chain in all cases, with a concomitant increase in NO_3_ consumption and NO production. These efflux pumps are proton/substrate antiporters, and their overexpression may lead to intracellular H^+^ accumulation, which may in turn offset the pH homeostasis. Indeed, all studied mutants showed a decrease in intracellular pH under anaerobic conditions. The fastest way to eliminate the excess of protons is by increasing oxygen consumption, a feature also displayed by all analyzed mutants. Taken together, our results support metabolic rewiring as a general mechanism to avoid the fitness costs derived from overexpression of *P. aeruginosa* multidrug efflux pumps. The development of drugs that block this metabolic “reaccommodation” might help in reducing the persistence and spread of antibiotic resistance elements among bacterial populations.

## IMPORTANCE

It is widely accepted that the acquisition of resistance confers a fitness cost in such a way that in the absence of antibiotics, resistant populations will be outcompeted by susceptible ones. Based on this assumption, antibiotic cycling regimes have been proposed in the belief that they will reduce the persistence and spread of resistance among bacterial pathogens. Unfortunately, trials testing this possibility have frequently failed, indicating that resistant microorganisms are not always outcompeted by susceptible ones. Indeed, some mutations do not result in a fitness cost, and in case they do, the cost may be compensated for by a secondary mutation. Here we describe an alternative nonmutational mechanism for compensating for fitness costs, which consists of the metabolic rewiring of resistant mutants. Deciphering the mechanisms involved in the compensation of fitness costs of antibiotic-resistant mutants may help in the development of drugs that will reduce the persistence of resistance by increasing said costs.

## INTRODUCTION

One largely accepted paradigm in the field of antibiotic resistance is that acquisition of resistance is associated with a fitness cost for the organism (1). In the case of mutation-driven resistance, the latter is usually achieved by mutations in genes encoding a number of elements, such as essential proteins (antibiotic targets) or transporters that are highly relevant for bacterial physiology. It is believed that mutations in such genes would cause “deadaptation” of bacteria to their environment: hence the less efficient resistant strains would be outcompeted by the more efficient susceptible ones in an antibiotic-free environment (2). Under this scope, antibiotic cycling strategies have been proposed to reduce the spread of resistance, with little success at the moment (3–5). A possible explanation for this failure is the fact that the fitness costs associated with antibiotic resistance are not derived from a global, nonspecific metabolic burden that would immediately reduce the ability of bacteria to proliferate in any ecosystem. Indeed, it has been reported that fitness costs depend on the mutation involved (6), with some resistance mutations occurring without any cost (7), that fitness costs can be gene and environment specific (8, 9), and that fitness costs can be compensated for by secondary mutations (10–14). Our group has proposed a new compensation mechanism that consists of the rewiring of bacterial metabolism (15). More recently, the possibility that the fitness cost of antibiotic resistance in mycobacteria can be compensated for by a nonmutational process has also been described (16). In view of the different effects that various resistance mechanisms have on bacterial physiology, it has been proposed that accurate estimations of fitness costs are needed for predicting the emergence of resistance (17–19) as well as for developing novel antibiotics (20).

Multidrug-resistant (MDR) efflux pumps constitute one of the most widespread resistance elements in bacteria (21–24). These determinants are generally chromosomally encoded and are highly conserved through different bacterial species (25–28). This suggests that they are ancestral elements present in bacteria long before the appearance of antibiotics and are involved in the extrusion of compounds other than clinically relevant antimicrobials (29). Bacterial MDR pumps have been grouped into five families: (i) the ATP-binding cassette (ABC) superfamily (30), (ii) the multidrug and toxic compound extrusion (MATE) family (31), (iii) the major facilitator superfamily (MFS) (32), (iv) the small multidrug resistance (SMR) family (33), and (v) the resistance/nodulation/division (RND) superfamily (34, 35). The RND pumps are most frequently found in Gram-negative bacteria (36). Efflux pumps are usually expressed at a basal level under laboratory growth conditions. Mutations in regulators and/or repressors result in overexpression of these elements, which in turn bring about antibiotic resistance (37–39). Twelve systems belonging to the RND family have been functionally described in *Pseudomonas aeruginosa* (23). Out of these, overexpression of MexAB-OprM, MexCD-OprJ, MexEF-OprN, or MexXY has been shown to play a relevant role in the acquisition of resistance (40, 41).

Fitness costs associated with the overexpression of efflux pumps could be the result of two aspects inherent in their mechanism: the constant extrusion of metabolic intermediates that are the natural substrates of the pumps and the energy expense brought about by the constant activity of the pumps in the presence of a substrate. Indeed, it has been shown that overexpression of MDR efflux pumps causes changes in bacterial physiology (15, 42, 43), including alterations in the infective capacities of different pathogens (43–47). Nevertheless, these putative fitness costs are not evident in every ecosystem. Previous results from our group have shown that overexpression of MexEF-OprN does not produce an unspecific fitness cost that would manifest itself as a reduced growth rate in rich medium (43). Instead, overexpression of this efflux pump causes profound changes in the *P. aeruginosa* physiology that interfere with the quorum sensing (QS) signaling pathway. Further work showed that MexEF-OprN overexpression is associated with a metabolic rewiring that includes increased expression of the nitrate respiratory chain (15). *P. aeruginosa* is a facultative anaerobic bacterium that can use nitrate as a final electron acceptor during anaerobic respiration (48). Expression of the nitrate respiratory chain requires low oxygen concentrations and the presence of nitrate in the growth medium (49, 50). Expression of this respiratory chain under aerobic conditions suggests that this shift is required to avoid incurring a fitness cost upon MexEF-OprN overexpression. Indeed, in the absence of nitrate, the mutant overexpressing MexEF-OprN is outcompeted by the wild-type strain. It remains to be elucidated whether this metabolic compensation is a general trait associated with the overexpression of RND efflux pumps or if it is MexEF-OprN specific. This is particularly relevant because MexT, a global regulator that might be involved in the regulation of the nitrate respiratory chain, also regulates MexEF-OprN expression (51–53). If this were the case, the observed metabolic compensation would be a side effect of the altered regulatory network displayed by this mutant and could not be generalized to other mutants overexpressing different RND efflux pumps.

We scrutinized mutants overexpressing MexAB-OprM, MexCD-OprJ, or MexXY for possible metabolic compensations. Our results show that a metabolic rewiring similar to that previously reported for the mutant overexpressing MexEF-OprN occurs in all mutants overexpressing these efflux pumps. This indicates that this is a feasible strategy to cope with the cost associated with the acquisition of antibiotic resistance by bacterial strains.

## RESULTS AND DISCUSSION

**FIG 1:**
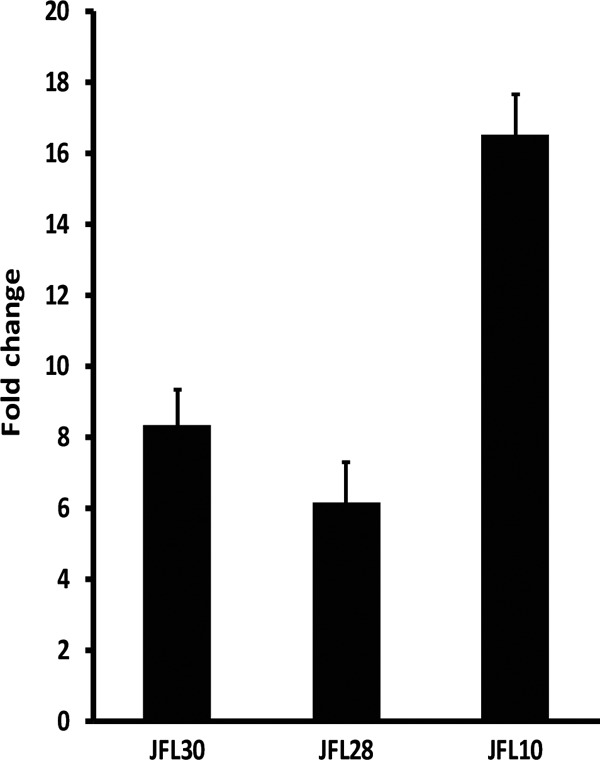
Relative expression levels of mutants overexpressing efflux pumps. The relative expression level of the studied efflux pumps in the overexpressing mutants in comparison with the wild-type strain was measured by real-time RT-PCR. JFL30 indicates relative expression of MexAB-OprM, JFL28 indicates relative expression of MexCD-OprJ, and JFL10 indicates relative expression of MexXY. The figure shows the means and standard deviations from three different experiments. In all cases, the differences between the wild type and the overexpressing strains were statistically significant (*P* < 0.05).

Previous work in our laboratory has shown that when *P. aeruginosa* overexpresses MexEF-OprN, the anaerobic nitrate respiratory chain is activated under aerobic conditions, and oxygen consumption increases (43). The energy needed for the RND pumps to function is generated by the proton gradient between the periplasm and the cytoplasm (35, 54). Increased expression of RND pumps might cause an increase in H^+^ concentration inside the cell, thus diminishing the internal pH. Indeed, *in vitro* studies using pumps collected in vesicles have shown that the constant activity of these systems may acidify the medium within the vesicle (55–58). Bacteria eliminate protons from the cytoplasm mainly through aerobic respiration (59). Oxygen uptake could conceivably be increased to cope with cytoplasmic acidification. This would result in a low intracellular oxygen concentration, which in turn would trigger the expression of the nitrate respiratory chain, as observed in our previous work (15). One particular aspect of MexEF-OprN is that the MexT global regulator controls its expression, in addition to that of several other genes (51–53). Moreover, uncontrolled expression of MexEF-OprN leads to the nonphysiological extrusion of kynurenine, an intermediate metabolite in the biosynthesis of the *Pseudomonas* quinolone signal (PQS), which is in turn a fundamental molecule in the QS regulatory system of *P. aeruginosa* (43). It is then possible that the observed metabolic rewiring is not a direct metabolic compensation, but rather an indirect consequence of the cross regulation of the nitrate respiratory chain when MexEF-OprN is overexpressed. We decided to study mutants overexpressing MexAB-OprM, MexCD-OprJ, or MexXY to determine whether this mechanism is a specific outcome of the MexEF-OprN-overexpressing mutant or constitutes a generalized response of *P. aeruginosa* mutants overexpressing RND efflux pumps. The mutants used in this study are *in vitro-*selected antibiotic-resistant mutants (Table 1). The selection method ensures that the mutants resemble those typically found in clinical settings. The RND efflux pumps are overexpressed as a consequence of mutations in genes encoding the local regulator of each specific efflux pump (42, 60). The relative level of expression of each efflux pump in the overproducing strains in comparison to the wild type is shown in Fig. 1.

**TABLE 1:** MICs of clinically relevant antibiotics toward *P. aeruginosa* mutants overexpressing efflux pumps

Strain	MIC (µg/ml)^a^						
	CIP	GEN	CHL	IMI	ATM	CAZ	KAN
PAO1-V	0.25	2	64	2	4	0.5	32
JLF30	1	2	512	2	32	0.5	24
JFL28	4	2	128	1	1	0.5	12
JFL10	4	8	256	2	4	1.5	64

a CIP, ciprofloxacin; GEN, gentamicin; CHL, chloramphenicol; IMI, imipenem; ATM, aztreonam; CAZ, ceftazidime; KAN, kanamycin.

### Increased expression of the nitrate respiratory chain is a general outcome for *P. aeruginosa* mutants that overexpress RND pumps.

**FIG 2:**
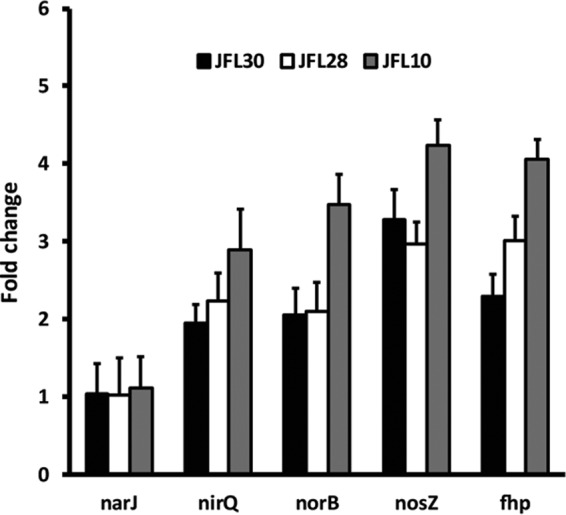
Expression of the nitrate respiratory chain in different *P. aeruginosa* mutants overexpressing RND pumps. The expression levels were obtained by real-time RT-PCR. As shown, the levels of expression are highest in the mutant JFL10 (gray bars), followed by JFL28 (white bars), and JFL30 (black bars). These results show that all mutants that overexpress RND pumps show increased expression of the nitrate respiratory chain under aerobic conditions. The figure shows the means and standard deviations from three different experiments. In all cases except for *narJ*, the differences between the wild type and the overexpressing strains were statistically significant (*P* < 0.05).

The expression of selected genes coding for enzymes involved in the denitrification process was measured by real-time reverse transcription (RT)-PCR in *P. aeruginosa* mutants that overexpress the RND pumps MexAB-OprM (JFL30), MexCD-OprJ (JFL28), and MexXY (JFL10) and in their wild-type counterpart (Fig. 2). Expression of a flavohemoprotein that functions in nitric acid detoxification under aerobic conditions was also measured. Increased expression of all operons of the nitrate respiratory chain was seen for all mutants in comparison with the wild-type strain (*P* < 0.05 in all cases), with the exception of *nar*. These results support the notion that overexpression of an RND pump itself, and not a specific regulatory pathway affected exclusively in the MexEF-OprN-overexpressing mutant, might activate alternative energy generation pathways in *P. aeruginosa*, such as the nitrate respiratory chain.

### Activation of the nitrate respiratory chain in mutants that overexpress RND efflux pumps increases nitrate consumption and nitric oxide production.

**FIG 3:**
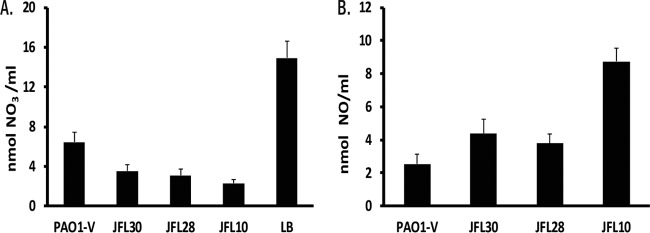
NO_3_ consumption and NO production in mutants that overexpress RND pumps. The NO_3_ (A) and NO (B) concentrations were measured using a nitric oxide colorimetric assay kit (Abcam, Inc., Cambridge, United Kingdom). After 24 h of culture, all strains had consumed the nitrate present in the LB medium (A). JFL10 consumes the largest amount of nitrate. These results correlate with the levels of nitric oxide production (B) as JFL10 also produces the largest amount of nitric oxide. These results indicate that the nitrate respiratory chain is active in all the mutants analyzed in this study. The figure shows the means and standard deviations from three different experiments. In all cases, the differences between the wild type and the overexpressing strains were statistically significant (*P* < 0.05).

Although we have observed that the expression of the genes encoding the nitrate respiratory chain is triggered in mutants overexpressing RND efflux pumps, this does not mean that the respiratory chain is effectively at work. It is known that activity of the nitrate respiratory chain leads to nitrate consumption and nitric oxide production (48). Although LB medium is not rich in nitrate, we have estimated its nitrate concentration to be 14 ± 2 µM (15). Nitrate consumption was measured after 24 h of culture in LB medium to determine whether the nitrate respiratory chain was active in the mutants overexpressing RND efflux pumps. As shown in Fig. 3A, JFL10 consumes the largest amount of nitrate after 24 h compared to the wild-type strain, followed by JFL28, and lastly JFL30. The differences are all statistically significant (*P* < 0.05 in all cases). The levels of nitric oxide production (Fig. 3B) are in agreement with these results, thus confirming that the mutants overexpressing RND efflux pumps have an active nitrate respiratory chain even when growing under aerobic conditions.

### The mutants overexpressing RND efflux pumps consume more oxygen than the wild-type strain without exhibiting a difference in growth rate.

The observed increase in the nitrate respiratory chain activity could be due to impairment in oxygen uptake. To test this possibility, the oxygen consumption of the different mutants was measured in the exponential and stationary growth phases (Table 2). All mutants consumed more oxygen than the wild-type strain in both growth phases (*P* < 0.05 in all cases), indicating that the cause of the activity of the nitrate respiratory chain was not impairment in oxygen uptake. The mutant that overexpresses MexXY (JFL10) has levels of consumption of 180% over that of the wild-type strain in the exponential phase and 130% over that of the other mutants. Similar results were observed in the stationary phase, in which JLF10 showed an oxygen consumption of 190% compared with the wild-type strain. Incidentally, this mutant exhibits the highest activity of the nitrate respiratory chain (see above). This further supports the hypothesis that both increased oxygen and nitrate respiration are required to compensate for fitness costs associated with the overexpression of RND efflux pumps. Indeed, despite the observed oxygen overconsumption, the doubling time of the different strains is similar to that of the wild-type strain (Table 3), suggesting that in a rich and aerated system, bacterial metabolic rewiring could compensate for the putative costs associated with overexpression of RND efflux pumps.

**TABLE 2:** Oxygen consumption of *P. aeruginosa* wild-type strain and resistant mutants

Strain	Mean ± SD oxygen consumption (nmol/g⋅min)^a^	
	Log phase	Stationary phase
PAO1-V	137 ± 14 (100)	110 ± 9 (100)
JFL30	189 ± 8 (138.2)	183 ± 11 (166.4)
JFL28	182 ± 9 (132.8)	181 ± 17 (164.5)
JFL10	254 ± 12 (185.4)	192 ± 6 (174.5)

a The percentage with respect to the wild-type strain PAO1-V is shown in parentheses.

**TABLE 3:** Growth rate of the *P. aeruginosa* wild-type strain and resistant mutants under different growth conditions

Strain	Mean doubling time (min) for:				
	20% oxygen^a^	1% oxygen^a^	0% oxygen^b^	Spent medium^a^	Spent medium + NO_3_^a^
PAO1-V	50 ± 3	57 ± 2	198 ± 6	59 ± 3	60 ± 4
JFL30	51 ± 2	58 ± 1	215 ± 7	58 ± 2	63 ± 5
JFL28	52 ± 3	66 ± 3	251 ± 9	75 ± 5	66 ± 4
JFL10	50 ± 4	70 ± 2	266 ± 4	78 ± 4	64 ± 3

a Growth in fermentor.

b Growth in Hungate tubes.

### Overproduction of RND pumps activates the expression of genes involved in anaerobic growth under low-oxygen-concentration conditions.

**FIG 4:**
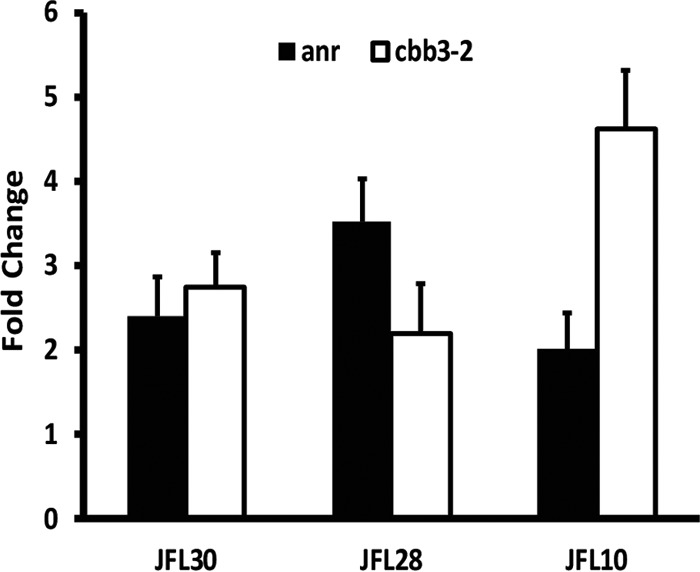
Effect of overexpression of RND efflux pumps on the expression of genes involved in microaerobic growth. The relative expression of *anr* (black bars) and *cbb_3_-2* (white bars) was analyzed under nonaerated conditions in a fermenter. The figure shows the ratio of expression between nonaerated and fully aerated conditions. For all mutants, increased expression of both genes can be seen under nonaerated conditions in the exponential phase. This phenomenon may be the result of a decrease in internal oxygen concentration in the bacterium due to overexpression of RND pumps. The figure shows the means and standard deviations from three different experiments. In all cases, the differences between the wild type and the overexpressing strains were statistically significant (*P* < 0.05).

*P. aeruginosa* activates the expression of a set of genes dedicated to life under microaerobic conditions when faced with low oxygen concentrations. One of the elements produced by these genes is the high-oxygen-affinity protein Cbb_3_-2, encoded by the PA1557-PA1555 operon. The ANR regulator controls the expression of *cbb_3_-2* (61, 62). The increased oxygen consumption observed in all of the mutants is likely to cause an intracellular low-oxygen environment where expression of *cbb_3_-2* and *anr* increases compared to that of the wild-type strain. To analyze this possibility, two sets of conditions were used: aerated cultures, with oxygen pumped along an airline at a constant concentration (20%), and nonaerated cultures, with oxygen derived only from the stirring process (15). As shown in Fig. 4, the expression of both genes is triggered in the mutants that overexpress the analyzed RND pumps (*P* < 0.05 in all cases), reaching a 3-fold increase of *anr* expression in the mutant JFL28, while this increase in expression was not observed in the wild-type strain. JFL10, the MexXY-overproducing mutant that exhibits the highest oxygen consumption among all mutants (see above), also exhibits the highest increase in *cbb_3_-2* expression. The observed changes in gene expression strongly suggest that the oxygen entering the cell is immediately consumed, most likely by reacting with the increased amount of intracellular H^+^ present in the cytoplasm due to the activity of efflux pumps. The activity of the efflux pumps as proton antiporters and the observed cytoplasm acidification (see below) require the presence of a substrate to be extruded. Since no external substrates were added in these experiments, our results strongly suggest that the studied efflux pumps have metabolic substrates that remain to be identified.

### The mutants overexpressing RND pumps show a decrease in intracellular pH under anaerobic conditions.

**FIG 5:**
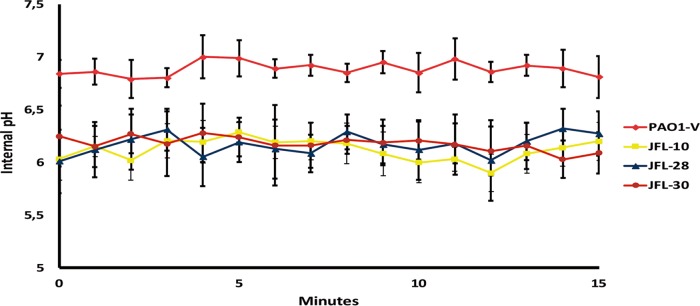
Overexpression of RND efflux pumps decreases *P. aeruginosa* intracellular pH under anaerobic conditions. Variations in internal pH under anaerobic conditions were measured for the wild-type strain PAO1-V and for the RND efflux pump-overexpressing mutants JFL30, JFL28, and JFL10 as described in Materials and Methods. For all mutants, a decrease in pH can be seen under anaerobic conditions compared to the wild-type strain. This result further supports the importance of oxygen availability for the elimination of the excess of protons generated by overexpression of RND pumps. The figure shows the means and standard deviations from three different experiments.

If the activity of RND efflux pumps produced an imbalance in the intracellular H^+^ concentration that is immediately compensated for by increased oxygen uptake, in the absence of oxygen, overexpression of such pumps should cause acidification of the cytoplasm. In agreement with this hypothesis, the mutants JFL10, JFL28, and JFL30 showed low internal pH when growing in the absence of oxygen (Fig. 5). These results further support the notion that the increased oxygen consumption of the mutants hereby analyzed constitutes a response to maintain the intracellular pH at a physiological level, thus alleviating the cost associated with the acquisition of antibiotic resistance and ensuring bacterial homeostasis.

### Limiting oxygen levels imposes a fitness costs for mutants overexpressing RND efflux pumps.

Our data suggest that oxygen overconsumption allows RND-overexpressing mutants to maintain their internal pH and their growth at the same level as the wild-type strain. We hypothesized that these mutants would exhibit a fitness cost under oxygen-limited conditions. To test this possibility, we calculated the growth rate of all mutants grown under low oxygen concentrations and in the absence of oxygen (Table 3). JFL10 and JFL28 showed a growth defect of over 10% under low oxygen concentrations. The defect was more pronounced in the total absence of oxygen, with a decrease of almost 20% compared with the wild-type strain. A growth defect was not so clear in the case of JFL30, since its behavior was very similar to that of the wild-type strain under low oxygen concentrations, while the growth rate exhibited a decrease of almost 3% in the absence of oxygen. These results further demonstrate the dependence on oxygen of these mutants when attempting to avoid the fitness cost caused by overexpression of the efflux pumps. It might be argued that overexpression of the nitrate respiratory chain itself is what causes the observed fitness cost; however, the fact that an increase in expression of these genes is a physiological response of wild-type cells during anaerobic respiration speaks against this possibility.

### The absence of nitrate, the electron acceptor of the *P. aeruginosa* anaerobic respiratory chain, unveils fitness costs in mutants overexpressing RND efflux pumps.

**FIG 6:**
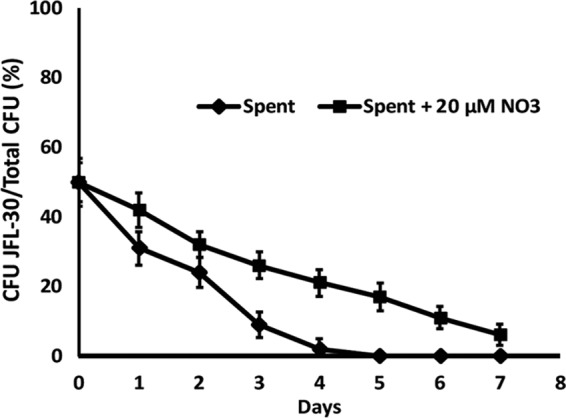
Growth competition between the wild-type strain and a mutant overexpressing MexAB-OprM in nitrate-free medium. The relative fitness between JFL30 and the wild-type strain was estimated by coculturing both strains and is represented as the percentage of mutant cells present at each time point (days). The absence of nitrate in the spent medium (solid line) produces a fitness cost in JFL30. However, the addition of nitrate to the medium partly reverts this cost. This result confirms the need for nitrate availability to avoid the fitness cost caused by overexpression of RND pumps. The figure shows the means and standard deviations from three different experiments.

Our work shows that increased oxygen respiration is required for compensation of the fitness costs associated with overexpression of RND efflux pumps and that the anaerobic respiratory chain is expressed and active in these mutants under aerobic conditions. However, it is unclear whether the activity of this anaerobic route of energy generation is also needed for compensation of the fitness cost or whether the observed increase in expression is merely a side effect of low intracellular oxygen levels. If the former were true, the mutants would be expected to exhibit growth impairment in the absence of nitrate. As in the case for oxygen-limiting concentrations, JFL28 and JFL10 showed growth problems in spent medium depleted of nitrate (15); the defect was partially compensated for when nitrate was added to the medium, whereas this defect was not so clear in the case of JLF30 (Table 3). To further determine whether or not JLF30 exhibited a fitness cost when growing without nitrate, competition tests of this mutant with its parental wild-type strain were performed. As shown in Fig. 6, after 4 days of competition JFL30 was displaced by the wild-type strain, thus demonstrating that overexpression of MexAB causes a fitness cost in nitrate-free medium. This result is partly reverted when nitrate is added to the medium, further demonstrating that nitrate is needed to compensate for the fitness costs associated with the expression of *P. aeruginosa* RND efflux pumps.

### Conclusions and perspective.

**FIG 7:**
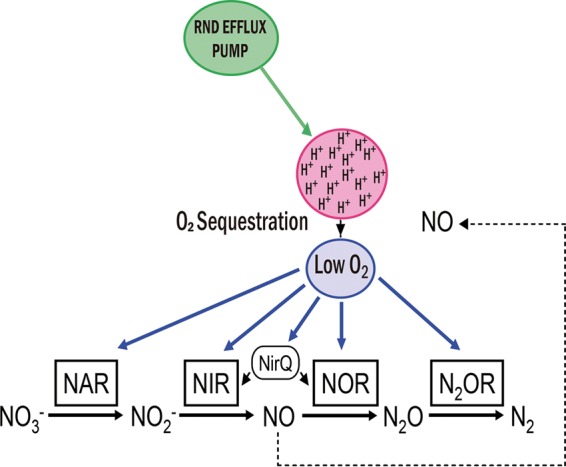
Model of the metabolic compensation associated with overexpression of RND efflux pumps. RND efflux pumps are proton antiporters. Overexpression of these elements could result in the accumulation of protons in the cytoplasm with a concomitant deleterious intracellular acidification. To cope with this problem, *P. aeruginosa* could make use of the intracellular oxygen to get rid of excess protons. Nevertheless, this oxygen sequestration might decrease intracellular oxygen tension in such way that bacteria sense the situation as a switch to oxygen-limited conditions. As a consequence, the expression of typical pathways of the anaerobic metabolism, such as the nitrate respiratory chain, is triggered. (This figure was inspired by reference 48.)

Overexpression of RND efflux pumps is among the main mechanisms of acquisition of multidrug resistance in *P. aeruginosa*. To date, 12 RND pumps have been described in this pathogen, but only four confer a clinically relevant antibiotic resistance: MexAB-OprM, MexCD-OprJ, MexEF-OprN, and MexXY. It is conceivable that overexpression of these elements may result in a fitness cost for the following reasons: First, efflux pumps are large tripartite protein complexes, and the energy required for their synthesis at nonphysiological levels might impair bacterial metabolism. Second, if an efflux pump extrudes metabolic intermediates or quorum sensing signals as it occurs for the MexEF-OprN (15) or MexAB-OprM systems (63), this could compromise bacterial physiology in selected ecosystems. Third, if an efflux pump substrate is present, the activity of the pump itself will consume bacterial energy. However, our results show that in a rich and oxygenated medium, overexpression of these efflux pumps does not impose a relevant fitness cost. RND efflux pumps are proton antiporters; consequently, their activity might alter the proton gradient since these pumps need a constant proton flow between the periplasm and the cytoplasm in order to function. A decrease in internal pH can compromise the bacterial physiology and lead to a fitness cost. The fastest way to eliminate protons is through aerobic respiration. We propose that this is the reason why mutants that overexpress RND pumps show higher oxygen consumption rates than their wild-type counterparts. Since oxygen is consumed to maintain the intracellular pH homeostasis, this overconsumption would not necessarily correlate with an increased amount of intracellular oxygen. Indeed, the fact that RND-overexpressing mutants present increased expression of the anaerobic respiratory chain even when *P. aeruginosa* is growing aerobically strongly suggests that overexpression of RND efflux pumps is probably generating intracellular low oxygen concentrations. It is important to mention that overexpression of the anaerobic respiratory chain is not just a futile consequence of a microaerobic cytoplasm among RND overproducers. The fact that in the absence of the NO_3_, the final electron acceptor of this respiratory chain, RND overproducers present a fitness cost indicates that the activity of the nitrate respiratory chain surfaces as an alternative for generating energy for *P. aeruginosa*. As shown in Fig. 7, altogether our data indicate that *P. aeruginosa* is capable of “reaccommodating” its metabolism in order to avoid the fitness cost generated by the acquisition of resistance due to overexpression of RND efflux pumps. One potential pitfall of the model is that the potential fitness costs we are tracking require not only overexpression of the efflux pump but also its activity, or in other words, the presence of a substrate. In this regard, it is important to recall that, although efflux pumps are capable of extruding antibiotics, they can also extrude several other compounds, some of which are bacterial metabolites (28, 29). Among them, it has been described that MexEF-OprN can extrude kynurenine (43) and that MexAB-OprN is able to extrude QS signals (63). The putative metabolic substrates of MexXY and MexCD-OprJ are not known, but our results strongly suggest that these efflux pumps are actively extruding some (for the moment ignored) metabolic substrates.

To summarize, our results indicate that metabolic rewiring is a feasible strategy to alleviate the fitness costs associated with the acquisition of antibiotic resistance. The identification of these metabolic changes might contribute to the development of drugs that increase the fitness cost of resistant pathogens, and this in turn may interfere with the onset of resistance and consequently the persistence of antibiotic resistance in the absence of selective pressure. In the case of *P. aeruginosa* RND overproducers, a drug capable of specifically inhibiting the nitrate respiratory chain without affecting the human terminal oxidases may be an effective solution to compromise bacterial physiology, thus increasing fitness costs associated with the acquisition of resistance.

## MATERIALS AND METHODS

### Strains and growth conditions.

The bacterial strains used in this study are described in Table 4. For all experiments, bacteria were grown in 25 ml of LB medium (Pronadisa) in 100-ml flasks with constant shaking at 250 rpm at a temperature of 37°C.

**TABLE 4:** Strains used in this study

Strain	Description	Source or reference
*P. aeruginosa*		
PAO1-V	PAO1 clinical isolate, wild type	42
JFL30	MexAB-OprM-hyperexpressing MDR mutant	42
JFL28	MexCD-OprJ-hyperexpressing MDR mutant	42
JFL10	MexXY-hyperexpressing MDR mutant	42
PT149	MexEF-OprN-hyperexpressing MDR mutant	60
*E. coli* TG1	Host for general cloning procedures	65

### Preparation of spent medium.

Nitrate-free medium was obtained as described in reference 15. Briefly, the PT149 strain that overexpresses MexEF-OprN was grown in LB medium for 24 h. The culture was centrifuged for 1 h at 8,000 × *g*, followed by centrifugation of the supernatant at 10,000 × g for a further hour at 4°C to completely remove the cells from the medium. In order to ensure optimal growth conditions, the pH of the medium was adjusted to 7.0 with a solution of 2 M NaOH. Finally, the supernatant was filtered through a 0.45-µm-pore filter, followed by a 0.22-µm-pore filter. This supernatant was incubated for 48 h at 37°C to demonstrate the complete absence of cells and discarded if any growth was detected.

### Growth in a fermentor.

In order to control the oxygen concentration in the culture, the different strains were grown in a 2.0-liter fermentor (BioStat MD) using a working volume of 1.5 liters. The initial inoculum was obtained from a bacterial culture grown in flasks overnight. The medium was inoculated at an initial optical density at 600 nm (OD_600_) of 0.01, and a stirring rate of 650 rpm was used. For aerated cultures, a constant airflow (20% oxygen) was used at a rate of 300 ml/min. The nonaerated cultures were just stirred, and no oxygen was added. Doubling times were estimated from independent cultures from 4 different days.

### Growth under anaerobic conditions.

The anaerobic nitrate respiration experiments were carried in 16- by 125-mm Hungate tubes capped with a septum and open-top screw caps (Bellco Glass, Inc.). In order to fully eliminate oxygen content from the liquid medium, N_2_ was bubbled through the medium for approximately 30 min in an anaerobic chamber, where the Hungate tubes were filled and capped, followed by sterilization in an autoclave. The nitrate needed to sustain growth under anaerobic conditions was obtained from a solution of 1 M KNO_3_, which was injected into each of the tubes at a final concentration of 10 mM. The initial inoculum for the assays under anaerobic conditions was obtained by seeding a single colony in 2.5 ml of LB medium and growth under regular aerobic conditions at 37°C overnight. In order to obtain an inoculum adapted to anaerobic respiration conditions, the Hungate tubes were injected with 100 µl of the overnight culture using a syringe and incubated at 37°C for 15 h without shaking. Finally, in order to conduct the experiments under anaerobic conditions, the preinoculum was injected to a final OD_600_ of 0.01. The cultures were incubated at 37°C without shaking. Both the sampling and the inoculation in the Hungate tubes were carried out using a syringe, and it was at these two points that the culture was gently mixed to homogenize the sample. For each sampling, 200 µl was taken to avoid excessive loss of culture.

### Estimation of oxygen consumption rate.

The oxygen consumption measurements were carried out using an Oxygraph oximeter (Hansatech) equipped with a Clark electrode. The mutants that overexpress the RND pumps and the wild-type strain were grown in 100-ml flasks with 25 ml of LB medium and incubated at 37°C with constant shaking at 250 rpm. Oxygen consumption was measured in the exponential phase (approximately 10^6^ CFU/ml) and the stationary phase (approximately 10^9^ CFU/ml). In order to measure the culture’s oxygen consumption, 2 ml was taken and placed into the oximeter to determine the respiration rate of the bacteria. The samples were then lyophilized to obtain the dry weight. The oxygen consumption rate is expressed as nanomoles of O_2_ consumed per minute per milligram (dry weight) of bacterial culture.

### Measurement of NO production and NO_3_^−^ consumption.

The nitric oxide colorimetric kit (Abcam, Inc., Cambridge, United Kingdom) was used to measure NO production and NO_3_^−^ consumption. Bacteria were cultured under aerobic conditions in 25 ml of LB medium at 37°C and shaking at 250 rpm. In order to take the measurements, 2 ml of culture was taken and centrifuged at 6,000 × *g* for 30 min. Each sample was then filtered through a 0.22-µm-pore filter. A total of 85 µl from each sample was used for the determination following the manufacturer’s instructions.

### Real-time RT-PCR.

The expression levels of the different genes of interest were measured by real-time RT-PCR. In the case of flask cultures, cells were collected in the logarithmic phase at an OD_600_ of 0.6, as described previously (43). For fermentor cultures, samples were collected at an OD_600_ of 0.8 for the logarithmic phase (15). Total RNA was isolated as described previously (43), using the RNeasy column system (Qiagen). A high-capacity cDNA reverse transcriptase kit (Applied Biosystems) was used to synthesize the cDNA. Fifty micrograms of the initially isolated RNA was used in each reaction. Three biological replicates, each with three technical replicates, were used in each of the assays. The relative levels of expression were calculated using the threshold cycle (2^−ΔΔ*CT*^) method (64). The expression of *rpsL* was used for normalizing the results. The primers used are listed in Table 5.

**TABLE 5:** Primers used in this study

Name^a^	Sequence
*nirQ*F	5′ GCGGTATCTGCTACCTGGAC 3′
*nirQ*R	5′ TAGGACACCACCAGCATGAA 3′
*norB*F	5′ CTACAACCCGGAAAACCTCA 3′
*norB*R	5′ AGCCACTTCTCGATCACCTC 3′
*nosZ*F	5′ ACGACGGCAAGTACCTGTTC 3′
*nosZ*R	5′ AGAACACGTAGCGGGTATGC 3′
*fhp*F	5′ ATTTCTACCGCACCATGCTC 3′
*fhp*R	5′ AGTTCCTGCAACTGGTCGAT 3′
*anr*F	5′ TGAAGAAAGGCGAATTCCTG 3′
*anr*R	5′ CGGATAGGTCTCGGTATCCA 3′
*ccoN2*F	5′ TCTACCACCTGATCCCGAAG 3′
*ccoN2*R	5′ CGACGAAGGAGTAGGTCAGG 3′
*rpsL*F	5′ GCAAGCGCATGGTCGACAAGA 3′
*rpsL*R	5′ CGCTGTGCTCTTGCAGGTTGTGA 3′

a F, forward; R, reverse.

### Measurement of internal pH.

The internal pH of the cell was measured as described in reference 15, using the fluorescent probe 5(6)-carboxyfluorescein diacetate succinimidyl ester (cFDASE). Briefly, cells were collected at an OD_600_ of 0.6 under aerobic and anaerobic conditions. The samples were centrifuged at 7,000 × *g* for 10 min at room temperature. The pellets were resuspended at a cell density of 10^5^ cells/ml in HEPES buffer (50 mM [pH 9.0]) containing 1 mM EDTA and incubated for 10 min at 30°C with 2 µM cFDASE. Following this incubation, the samples were washed twice and resuspended in phosphate-buffered saline (PBS) buffer (pH 7.0) containing 1 mM MgCl_2_. In order to eliminate excess from the nonconjugated probe, the samples were treated with 10 mM glucose in PBS. Cells were then centrifuged at 7,000 × *g* for 10 min, and the pellets were washed twice and resuspended in a buffer composed of 50 mM Tris, 50 mM morpholineethanesulfonic acid (MES), 140 mM choline chloride, 1 mM MgCl_2_, 10 mM KCl, 10 mM NaHCO_3_, and CaCl_2_ (pH 7.0). One hundred fifty microliters of each sample was placed on black 96-well plates (Nunclon Delta Surface) and incubated at 30°C in a spectrophotometer (Infinite 200; Tecan). For the anaerobic conditions, the plates were incubated in an anaerobic jar, where the oxygen was eliminated using the AnaeroGen atmosphere generation system (Oxoid). All experiments were carried out in triplicate.

### Competition experiments.

The competition experiments were carried out as described in reference 43. Briefly, the strains PAO1-V and JFL30 were grown individually overnight at 37°C with shaking at 250 rpm. The competition began when the two strains were mixed in a single flask containing 25 ml of LB medium free of antibiotics. The mixed cultures were incubated for 24 h at 37°C. A new flask containing fresh medium was then used to reseed the mixture with a dilution of 1:1,000. In order to differentiate between JFL30 and the wild-type strain PAO1-V, serial dilutions of the culture were placed on plates in LB agar and in LB agar with 64 µg/ml of tetracycline. The results were expressed as the percentage of resistant cells versus the total number of cells.

### Statistical analysis.

The results were analyzed using the Student *t* test to determine their significance.
